# The first-year growth response to growth hormone treatment predicts the long-term prepubertal growth response in children

**DOI:** 10.1186/1472-6947-9-1

**Published:** 2009-01-12

**Authors:** Berit Kriström, Jovanna Dahlgren, Aimon Niklasson, Andreas FM Nierop, Kerstin Albertsson-Wikland

**Affiliations:** 1 Göteborg Pediatric Growth Research Center, Institute for Clinical Science, The Sahlgrenska Academy at University of Gothenburg, SE-416 85 Gothenburg, Sweden; 2 Department of Clinical Science/Paediatrics, Umeå University, SE-901 87 Umeå, Sweden; 3 Muvara, Tijmtuin 8, 2353 PH Leiderdorp, The Netherlands

## Abstract

**Background:**

Pretreatment auxological variables, such as birth size and parental heights, are important predictors of the growth response to GH treatment. For children with missing pretreatment data, published prediction models cannot be used.

The objective was to construct and validate a prediction model for children with missing background data based on the observed first-year growth response to GH. The accuracy and reliability of the model should be comparable with our previously published prediction model relying on pretreatment data. The design used was mathematical curve fitting on observed growth response data from children treated with a GH dose of 33 μg/kg/d.

**Methods:**

Growth response data from 162 prepubertal children born at term were used to construct the model; the group comprised of 19% girls, 80% GH-deficient and 23% born SGA. For validation, data from 205 other children fulfilling the same inclusion and treatment criteria as the model group were used. The model was also tested on data from children born prematurely, children from other continents and children receiving a GH dose of 67 μg/kg/d.

**Results:**

The GH response curve was similar for all children, but with an individual amplitude. The curve SD score depends on an individual factor combining the effect of dose and growth, the 'Response Score', and time on treatment, making prediction possible when the first-year growth response is known. The prediction interval (± 2 SD_res_) was ± 0.34 SDS for the second treatment year growth response, corresponding to ± 1.2 cm for a 3-year-old child and ± 1.8 cm for a 7-year-old child. For the 1–4-year prediction, the SD_res _was 0.13 SDS/year and for the 1–7-year prediction it was 0.57 SDS (i.e. < 0.1 SDS/year).

**Conclusion:**

The model based on the observed first-year growth response on GH is valid worldwide for the prediction of up to 7 years of prepubertal growth in children with GHD/ISS, born AGA/SGA and born preterm/term, and can be used as an aid in medical decision making.

## Background

In a clinical setting, when considering prescribing growth hormone (GH) treatment for a short or slowly growing child, tools that reliably predict the growth response to treatment are welcome. Published prediction models rely on pretreatment auxological variables and often on parental heights [1-5], variables that are often impossible to obtain. In reality, missing data on, for example, birth size and gestational age, may also complicate the decision of what disease-specific model to use.

Most prediction models give information on the estimated growth response for only the first or the first 2 years of treatment [1-5]. Growth response on treatment is positively influenced by a young age at the start of treatment [1-6] and, as height at onset of puberty highly influences final height [7], a model for prediction of not only annual but long-term prepubertal growth response on GH treatment would be a valuable contribution to the treatment decision-making process.

The aim of this project was to develop a prediction model for growth in response to GH in prepubertal children, relying only on data that could be obtained in different health settings, and also in adopted and immigrant children (i.e. where information on birth size, gestational age, growth during early life and parental heights are missing). However, accuracy and reliability should not be reduced compared with our previously published prediction model relying on pretreatment data [2,5].

It is an empirical finding that first year growth in response to GH is an indicator of the growth response in subsequent years of treatment, a finding confirmed in models predicting growth response on a yearly basis in children with GH deficiency (GHD) [1,2,5] or non-GH-deficient short children born small for gestational age (SGA) [3,5] or appropriate for gestational age (AGA; children with ISS) [2,5,6]. However, an accurate model for predicting long-term growth in response to GH in children with missing birth data has not yet been presented.

The hypothesis in the present study was that the observed first-year growth response to a certain dose of GH in prepubertal children would provide enough information with which to construct a prediction model with a model error low enough to be useful in clinical practice for the prediction of future prepubertal growth response on GH treatment. By this, it should be possible to individualize the GH dose and thereby the growth outcome until the onset of puberty.

## Patients and Methods

### Patients

Growth response data until puberty were used from prepubertal children on GH treatment in Sweden retrieved from the National Registry or from clinical trials. Every 3 months, the dose of GH was adjusted according to weight, height was measured with a standardized technique, and pubertal status and compliance were checked.

### Model group

Growth data during GH treatment were obtained from 162 prepubertal children born at term (19% girls; 80% GH-deficient; 23% born SGA) and for whom 2 years or more of prepubertal growth data were available and age was 2.5 years or more at the start of treatment (Table 1). The model group in this paper is a subset of the group of patients modeled in a previous study [2], having at least one measurement after 1 year of treatment and a second after 2–7 years of treatment. An arginin-insulin tolerance test (AITT) was used to confirm diagnosis with GHD or ISS (cut off, the 'old 10 μg/L') [8]. GH dose was approximately 33 μg/kg/d (Table 1). No child with combined pituitary insufficiency or chronic disease potentially affecting growth was included. Children with a recognized syndrome were also excluded, apart from a few children with Silver-Russell syndrome who had a birth length or weight below -2 SDS [9].

**Table 1:** The characteristics of the children included in the model group (n = 162) and in the validation group (n = 205).

	Model group (30 girls, 132 boys)				Validation group (58 girls, 147 boys)			
Variables	Median	Min	Max	n	Median	Min	Max	n

**At birth**								
Gestational age (weeks)	40	37	42	162	40	37	42	205
Height at birth SDS	-1.07	-2.65	2.6	162	-1.48	-5.75	1.9	205
Weight at birth SDS	-0.88	-2.75	3.24	162	-1.28	-4.5	1.81	205
**At GH start**								
Age at start of GH treatment (yrs)	8.3	2.78	13.92	162	7.78	2.51	12.53	205
Height SDS	-2.91	-4.59	-1.54	162	-2.84	-4.76	-1.29	205
Weight SDS	-2.58	-4.29	0.73	162	-2.5	-5.28	1.28	205
Weight for height SDS	-0.54	-3.31	3.85	162	-0.61	-3.08	4.09	205
Body mass index (kg/m^2^)	15.4	12.88	23.98	162	15.19	12.25	23.76	205
Change in height SDS during pre-treatment year	0.02	-0.85	0.58	144	0.01	-0.71	0.68	186
Target height SDS	-0.75	-2.4	1.26	162	-0.66	-2.71	0.94	205
Fathers height SDS	-1.13	-4.93	1.91	162	-0.83	-3.86	2.02	205
Mothers height SDS	-1,25	-3.55	1.22	162	-1.25	-3.55	1.55	205
Diff MPH SDS	-2.26	-4.98	-0.8	162	-2.27	-5.11	-0.41	205
GH_max _during AITT (mU/L)	19.8	1.7	229.4	160	21	2.4	124.88	181
GH_max _of 24 h profile (mU/L)	34.65	3.88	81.08	64	32.8	7.55	235.37	94
IGF-I at GH start	96.25	11.68	219	68	80	8	330	150
IGF-I SDS	-0.6	-6.69	1.38	68	-1.1	-7.43	3.03	150
GH dose (ug/kg.day)	0.033	0.027	0.04	162	0.033	0.023	0.043	205
**During treatment**								
Change in height SDS first yr	0.72	0.3	2.26	162	0.74	0.07	2.56	205
Change in height SDS during 2 yrs	1.15	0.4	2.88	162	1.14	0.23	3.12	205

Growth hormone (mU/L) levels were analysed at GP-GRC, GU, with Delfia monoclonal assay, Wallac, Finland, standard WHO IRP 80/505, [8].

Insulin-like growth factor I (IGF-I) (μg/L) were analysed according to Blum and Breier [26] and transformed into SD scores (SDS) according to gender and age of our prepubertal reference [27].

### Validation group

Data from 205 children (28% girls; 74% GH-deficient; 43% born SGA), fulfilling the same inclusion and treatment criteria as the model group, were used to validate the model (Table 1).

### Test groups

#### Children born prematurely

The model was applied to data from a separate group of 19 children born prematurely with a gestational age of 30–36 weeks (68% SGA) and given GH at a dose of 33 μg/kg/d (Table 2).

**Table 2:** The characteristics of the children included in the test groups of children born prematurely (n = 19) and in children from other continents (n = 78).

	Preterm (6 girls, 13 boys)				From foreign continents (27 girls, 51 boys)			
Variables	Median	Min	Max	n	Median	Min	Max	n

**At birth**								
Gestational age (weeks)	35	32	36	19	38.5	27	41	28
Height at birth SDS	-2.55	-8.09	1.43	19	-1.76	-3.91	0.38	6
Weight at birth SDS	-2.25	-6.12	0.11	19	-1.27	-3.95	0.63	8
**At GH start**								
Age at start of GH treatment (yrs)	6.38	3.02	10.1	19	8.35	3.13	13.47	78
Height SDS	-2.81	-4.08	-2.01	19	-3.27	-6.31	-0.74	78
Weight SDS	-2.78	-5.15	-1.56	19	-3.11	-5.66	1.12	78
Weight for height SDS	-0.97	-3.29	0.71	19	-0.71	-3.8	4.23	78
BMI (kg/m^2^)	14.5	12.18	16.5	19	15.35	11.49	22.23	78
Change in height SDS during pre-treatment year	0	-0.45	0.52	17	0.07	-0.73	0.59	69
Target height SDS	-0.58	-1.89	1.26	19	-1.23	-3.72	0.5	39
Father height SDS	-1.13	-3.41	1.3	19	-2.04	-4.62	0.54	39
Mother height SDS	-0.84	-3.39	1.55	19	-2.07	-5.85	0.4	41
Diff MPH SDS	-2.23	-3.43	-1.27	19	-1.76	-4.35	1.27	39
GH_max _during AITT (mU/L)	24.15	11	70.4	18	20.72	1.09	135.57	30
GH_max _of 24 h profile (mU/L)	19.95	14.3	48.05	11	42.69	12.37	88.79	43
IGF-I at GH-start	60	19	187	18	78	12	259	19
IGF-I SDS	-1.57	-5.42	0.72	18	-1.35	-7.02	1.75	19
GH dose (ug/kg.day)	0.037	0.027	0.043	19	0.033	0.023	0.047	78
**During treatment**								
Change in height SDS first yr	0.67	0.47	1.16	19	0.68	-0.1	1.74	78
Change in height SDS during 2 yrs	0.92	0.63	1.69	19	1.06	0.11	2.79	78

#### Children from non-European continents

Growth data for 78 children from non-European continents (four from Africa, 24 from Asia, 24 from other continents and 26 who had parents from different continents) given GH at a dose of 33 μg/kg/d were also tested in the model. Out of these 78 children, 28 had a diagnosis of GHD and 24 a diagnosis of ISS; in 26 children a GH-provocation test had not been performed. Birth data were missing in the majority of these patients (Table 2).

#### Children treated with a higher GH dose

In order to study the robustness of the model, growth data from 15 children treated with a GH dose of 67 μg/kg/d, but otherwise fulfilling all inclusion criteria for the model, were assessed.

### Methods

#### Auxology

Birth weight SDS and birth length SDS used in the algorithms were calculated according to Niklasson et al. [10,11]. For the prepubertal growth SDS calculation we used the formula of the childhood component [12] of the current Swedish growth references [13].

#### Curve fitting for a non-linear growth-response curve

During our earlier work for the prediction of the 0–1 year and 0–2-year growth responses to GH treatment in prepubertal children with GHD and ISS, models were constructed based exclusively on data that could be obtained from before the start of GH treatment, heavily relying on auxological data at birth and during the first 2 years of life and parental heights [2].

Measurements of growth response were obtained at different treatment times; for adequate correction of these differences a non-linear growth-response curve was computed by curve-fitting. It was based on observed longitudinal measurements from a subset of the model group of patients reported previously [2] during 1–7 prepubertal-years of GH treatment. This subset of the model group was used in the previous study [2] to establish the formula Δheight SDS_Pred_(t) = F_a_(t)+M*F_b_(t), where the individual parameters, M, and the form of the curves, F_a_(t) and F_b_(t), are fitted with alternating least squares. For fixed curves F_a _and F_b_, each individual parameter, M, was fitted with the regression y = xb+a, with regression weight b = M, intercept a = 0, response y = delta ht SDS(t) - F_a_(t) and predictor x = F_b_(t) for all observed treatment times t ≥ 0. In this paper, the same model group was used to estimate each individual regression weight, b = M, for the (fixed) response curve using only two measurement points, t = 0 and t = 1, resulting in a curved regression line that can be extrapolated after 1 year of treatment. In this way, the extrapolated response curve can be applied as a prediction model at 1 year of treatment, aiming to avoid the need for pretreatment auxology data.

**Figure 1:**
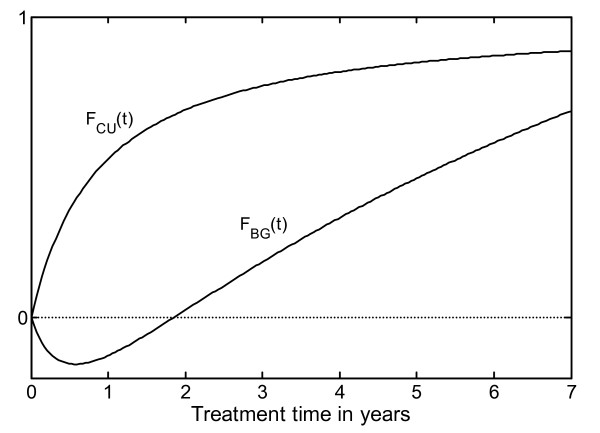
**Graphic illustration of the two equations that, when combined, produce the non-linear growth response curve**. The hyperbolic equation F_CU_(t) can be regarded as a 'catch-up' equation, (by multiplying with the individual parameter M_Model_) asymptotically modulating individual amplitude with time. The "1" on the y-axis gives the asymptotic value of the F_CU_(t), and the Equation F_BG_(t) can be interpreted as a 'baseline growth' equation that introduces a delayed general growth response with a greater relative impact on individuals with low levels of catch-up growth than on individuals with high levels of catch-up growth.

Furthermore, the old complex equations, F_a_(t) and F_b_(t), were reparameterized to facilitate more simple clinical interpretation, especially for equation F_b_(t) (Figure 1).

Our previous equation, F_a_(t) [2], was replaced by the equation_Basic Growth_: F_BG_(t) = ^e^log (1 + t*(0.205 - 1/(1 + 2.1*t))) and the equation F_b_(t) [2] was replaced by the equation_Catch Up_: F_CU_(t) = t/(t + 0.894), which is an asymptotic catch-up function, whereas F_b_(t) is not.

The accuracy of this reparameterization was assessed by evaluating the maximum absolute deviation between the old [2] and new growth response curves. For all theorical values of M between 0 and 5, which is the biological observed range, the maximum absolute deviation was 0.01 SDS over the first 4 years of treatment and 0.1 SDS over the remaining 5–7 years of treatment. The maximum deviation is much smaller than the measurement error.

The individual growth-response curves can be computed by adding the two equations, where only F_CU_(t) is multiplied by an individual factor, a model parameter M_Model _[2]. This individual model parameter is dependant on the GH dose (here 33 μg/kg/d) and the individual growth response. The parameter M_Model _was predicted for the individual child in our previous work [2] from pretreatment variables according to background and investigational data. The resulting predicted individual growth response was given in the algorithm by equation: Δheight SDS_Pred_(t) = F_BG_(t) + M_Model _*F_CU_(t).

#### The clinical concept 'Response Score' (RS)

The M_Model(Individual) _*F_CU_(t) part of the equation provides an individual predicted catch-up growth curve over the prepubertal years, approaching an asymptotic value (Figure 1). This gives a value for the mathematical catch-up level for each individual child, expressed by the GH growth-response equation: RS_pred _= M_Model _*F_CU_(t = 8) where time 't' is approaching infinity and F_CU_(t = 8) = 1.

**Figure 2:**
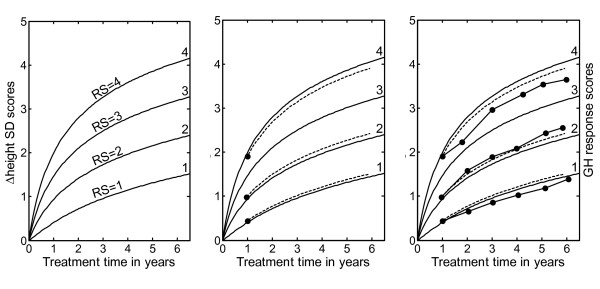
**GH response chart for visualization of Response Scores (RS) on different levels (i.e. growth responses for children with variable individual responsiveness to GH)**. Left panel: The RS chart is given with 'isolines' ('channels') for RS = 1, 2, 3 and 4 which is indicated in the figure. For the individual child at treatment time 1 year (x-axis), the observed growth response (Δheight SDS) is found on the y-axis to the left and following the corresponding curve the individual RS can be found to the right. Middle panel: Observed first-year Δheight SDS on treatment for three prepubertal children from the validation group (filled circles). The individual calculated Response Score (dotted line) is found on the right axis. The function for the Response score is: RS(t) = (Δheight SDS_obs _- F_BG_(t))/F_CU_(t), where F_BG_(t) = ^e^log (1 + t*(0.205 - 1/(1 + 2.1*t))) and F_CU_(t) = t/(t + 0.894); t = GH treatment time in years. Right panel: Individual Response Scores from the same three children, based on observed Δheight SDS on treatment at different time points (filled circles at treatment time 2, 3, 4, 5 and 6 years) in comparison with the predicted (dotted line). Response Score was consistent over time within an individual child, and the inclusion in the model of measurements made later than 1 year after the start of GH treatment are acceptable.

The total growth response during time 't' can be expressed by the growth response equation: Δheight SDS (t) = F_BG_(t) + RS_pred _* F_CU_(t) (Figure 2 left panel). A general "basic growth" function (F_BG_(t)) is added to a general "Catch-Up" function (F_CU_(t)) with an individual catch-up level (RS_pred_), which can be monitored or predicted. The response score gives a measure of growth in response to GH, independent of treatment duration.

#### Using the observed Response Score (RS) for prediction

For each observed measurement of individual growth response on GH treatment (Δheight SDS_obs_), it is possible to estimate the individual RS by defining the following Response Score equation: RS(t) = (Δheight SDS_obs _- F_BG_(t))/F_CU_(t) (Figure 2 middle panel). Thus, the RS can be estimated with only four variables: height at the start of treatment and after at least 1 year of GH treatment [14], gender and age. These variables are used to compute the Δheight SDS_obs _^1 yr ^and produces an individual RS^1 yr^. Using the RS^1 yr^, the Δheight SDS(t) on GH treatment over time (t) can be predicted with the model provided that the child remains prepubertal.

This gives the prediction model: Δheight SDS(t) = F_BG_(t) + RS^1 yr ^* F_CU_(t)

where F_BG_(t) = ^e^log (1 + t*(0.205 - 1/(1 + 2.1*t))) and F_CU_(t) = t/(t + 0.894); t = GH treatment time in years.

For each child, the RS(t) value that is calculated from an observed change in height SDS (Δheight SDS^t^_obs_) should remain constant during GH treatment, as long as no concomitant distress interferes with growth.

### Ethics

The study was approved by the Ethics Committee of the Medical Faculties of the Universities of Göteborg, Lund, Linköping, Uppsala and Umeå and of the Karolinska Institute. Informed consent was obtained from the parent(s) or person(s) with custody of the child and from the child, where appropriate.

## Results

### Observed response score for prediction

Figure 2 right panel shows the observed growth response, for comparison with the predicted growth response made after 1 year on treatment, on the four variables: age, gender, height at start of treatment and height after 1 year on GH treatment.

### Validation of long-term growth in response to GH on data from children born AGA/SGA at term

The observed individual growth responses to GH in 205 children during 1–7 years of prepubertal growth were used to validate the predictions made based on the observed first-year growth and the corresponding Response Score. The validation group was examined both in total and subdivided into children born AGA and SGA; data are presented in Table 3 together with corresponding data from the group used to construct the model. The SD_res _of the validation group is as low as for the model group, indicating that the model gives an accurate estimation of the individual growth response curve for the 1–7 years of prepubertal growth on GH treatment. The SD_res _was 0.17 SDS for 1–2 years of treatment and 0.57 SDS for the total period of 1–7 years of treatment (i.e. < 0.1 SDS/year during 6 years of GH treatment). A separate analysis showed no significant difference between the growth response to GH treatment in children born AGA and children born SGA (Table 3).

**Table 3:** Model error for the prediction of the 1–7-year GH growth response expressed in Dheight SD scores for the model group and the validation group separately: for the total group and subdivided into children born AGA or SGA.

Model group				Validation group			
Total group:	**n**	**SDres**	**SDstudRes**	Total group:	**n**	**SDres**	**SDstudRes**

1–2 year	162	0.17	1.00	1–2 year	205	0.19	1.11
1–3 years	97	0.29	1.00	1–3 years	111	0.28	0.97
1–4 years	53	0.38	1.00	1–4 years	58	0.35	0.90
1–5 years	34	0.43	1.00	1–5 years	40	0.45	1.04
1–6 years	17	0.48	1.00	1–6 years	29	0.40	0.84
1–7 years	7	0.57	1.00	1–7 years	9	0.43	0.75

AGA:				AGA:			
1–2 year	125	0.17	0.99	1–2 year	117	0.19	1.12
1–3 years	69	0.28	0.96	1–3 years	63	0.26	0.90
1–4 years	39	0.39	1.02	1–4 years	31	0.31	0.82
1–5 years	21	0.44	1.03	1–5 years	18	0.39	0.92
1–6 years	8	0.45	0.95	1–6 years	15	0.42	0.88
1–7 years	7	0.57	1.00	1–7 years	3	0.26	0.45

SGA:				SGA:			
1–2 year	37	0.18	1.05	1–2 year	88	0.19	1.09
1–3 years	28	0.31	1.08	1–3 years	48	0.30	1.05
1–4 years	14	0.36	0.93	1–4 years	27	0.37	0.98
1–5 years	13	0.41	0.96	1–5 years	22	0.49	1.13
1–6 years	9	0.50	1.04	1–6 years	14	0.37	0.79
1–7 years	0	-	-	1–7 years	6	0.49	0.87

SDres = root mean square error of the residuals.

SD stud Res = SDres/SDresModelgroup

For a reliable model, the SD_res _should be similar, or lower, for the validation group compared with the SD_res _from the model group.

### Group validation of estimated Response Scores

**Figure 3:**
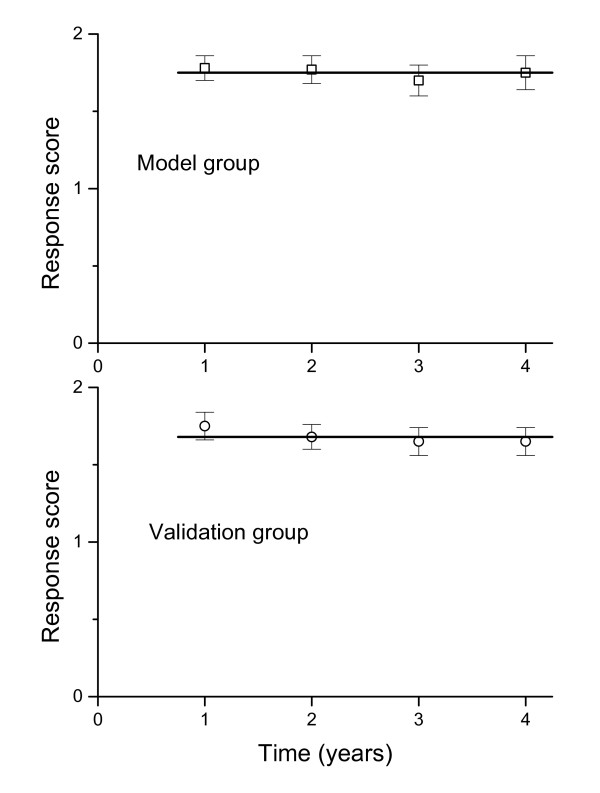
**The group mean values ± SEM for the Response Score calculated at 1, 2, 3 and 4 years of GH treatment**. The equation was applied to data from children who have values from all four annual measurements on GH treatment: the model group (n = 51) and validation group (n = 54). The observed height at the yearly measurement is used for estimation of the RS (1–4 years). This shows that a reliable model gives constant RS values during 4 years of GH treatment.

The values for the Response Scores over treatment time t (RS(t)) were calculated for 51 of the 162 children on GH treatment in the model group for whom prepubertal growth measurements were available at precise annual intervals during the first 4 years of treatment (Figure 3). When the RS equation was applied (i.e. validated) on data from 54 children from the validation group fulfilling the same criteria, the mean RS values remained constant as for the model group (i.e. the results were consistent).

The similarity with respect to mean growth in response to GH over all years shows that the model and validation groups are clinically comparable with respect to the average growth response; the difference between group mean yearly changes in height SDS is a maximum of 0.02 SDS. The model and validation groups were also studied after subdivision into quartiles according to the RS^1 yr ^level; the mean and range at each yearly measurement remained the same for each quartile (data not shown).

### Test on data from children born prematurely

The prediction model based on RS^1 yr ^was tested on a separate group of 19 children born prematurely at a gestational age of 30–36 weeks, Table 4. The diminishing number of children during the treatment period reflects the numbers remaining prepubertal. The SD_res _are consistent with the results for the children born at term. Dividing the prematurely born children into AGA and SGA groups gave very small numbers, but there was no difference in the SD_res _for the two groups.

**Table 4:** Test of growth in response to GH treatment in different cohorts of children: born preterm from other countries or treated with 67 μg/kg/d of GH in children born preterm or at term.

Children born preterm (30–36 w) GH dose 33 μg/kg/d			
	**n**	**SDres**	**SDstudRes**
1–2 year	19	0.16	0.93
1–3 years	15	0.31	1.07
1–4 years	7	0.44	1.15
1–5 years	5	0.27	0.63
1–6 years	4	0.51	1.07
1–7 years	2	0.47	0.83

			

**Children from other continents** GH dose 33 μg/kg/d			

	**n**	**SDres**	**SDstudRes**
1–2 year	78	0.18	1.07
1–3 years	42	0.25	0.88
1–4 years	20	0.35	0.9
1–5 years	10	0.34	0.79

			

**GH dose of 67 μg/kg/d** Born at term			

	**n**	**SDres**	**SDstudRes**
1–2 year	15	0.19	1.13
1–3 years	7	0.31	1.09
1–4 years	2	0.37	0.95

			

**GH dose of 67 μg/kg/d** Born preterm			

	**n**	**SDres**	**SDstudRes**
1–2 year	7	0.17	1.01
1–3 years	2	0.26	0.90

SDres = root mean square error of the residuals.

SD stud Res = SDres/SDresModelgroup

### Test on data from children coming from other continents

The prediction model was tested, and found to be valid on growth data from 78 children from other continents who seldom had complete data on birth size, pre-treatment growth and/or parental heights (Table 4).

### Test on data from children treated with an increased GH dose

In order to study the robustness of the model for differences in GH dosing, data from children fulfilling the inclusion criteria but being treated with a higher GH dose (67 μg/kg/d) were checked in the prediction model. The results are presented in Table 4; the SD_res _was of the same low magnitude as for children treated with the lower GH dose, showing that the model may also be applicable in such patients.

## Discussion

The hypothesis for the present study was confirmed, i.e. the observed first-year growth response on GH treatment contained enough information to be used for prediction of up to 7 years of prepubertal growth on GH treatment with a model error below 0.1 SDS/year for the entire 7-year period. The validated model is accurate and reliable and only needs information on four variables: gender, age, height at start of GH treatment, and observed first-year growth response to GH. This information is easily obtained in a clinical setting. The model can also be used for the children of immigrants for whom early growth data and parental height data are missing, as no pre-treatment investigations are necessary. Information on first-year growth in response to GH treatment makes it possible to estimate an individual Response Score, which can be used in the model in place of the pre-treatment data used in our previous prediction models to give equally accurate growth predictions [2-5].

A prerequisite for construction of the model is that we can demonstrate that the growth response to GH treatment can be fully described by a common equation for prepubertal non-syndromic children, clinically classified as GH-deficient/ISS, AGA/SGA and born later than 30 weeks of gestation. Using growth-response equations which are modulated by an individual parameter depending on individual responsiveness and GH dose, we show that the model works on empirical data from an independent group of 205 children fulfilling the inclusion criteria for the model group and also on data from more than 100 children with short stature as a result of other diagnoses. Thus, the present model was demonstrated to be valid on independent data from children classified as GH-deficient/ISS, born AGA/SGA, born at term/preterm (> 30 weeks gestational age), of Swedish or non-Swedish origin and receiving a GH dose close to 33 ug/kg/d or 67 ug/kg/d. Although these results are valid for the model, the groups of children treated with higher dose or born preterm are limited in number and data from more children are needed for further validation.

The performance of the present model, demonstrated by the SD_res _for the second year, is 0.17 SDS, corresponds to a 95% prediction interval of ± 0.34 SDS with very similar results for the model group and the validation group. This gives a second year prediction interval of ± 1.2 cm for a 3-year-old child and ± 1.8 cm for a child aged 7 years. For 1–4 years of treatment, the SD_res _is as low as 0.38 SDS (i.e. 0.13 SDS/year) and for the period from 1–7 years, the SD_res _is 0.57 SDS (i.e. < 0.1 SDS/year). Thus, knowing only the first-year growth response to GH treatment makes it possible to predict the total growth response for up to 7 prepubertal years (i.e. until the start of puberty). This is of great clinical significance as height at the onset of puberty has an important influence on adult height [7,8,15-18]. In clinical practice, information on growth response can be used to adjust the GH dose and by that improve prepubertal height outcome.

In this project a new concept was introduced: the individual Response Score at 1 year which is dependent on both the GH dose and the GH responsiveness of the child. For each child, it is now possible to calculate an individual Response Score value using the observed change in height SDS during a given treatment time period. Due to seasonal variation in both spontaneous growth and growth during GH treatment [14,19], the recommendation must be to allow a full year of growth for use in the prediction model. A height measurement obtained at more than 12 months of treatment will still be useful for calculation of the Response Scores, due to the growth response curve being non-linear. As the observed height will be very important for the accuracy of the prediction, measurement error should be minimized by using a standardized measuring technique, with measurements preferably performed by the same person and at the same time of the day. Using the obtained growth response^t ^and thereby the estimated RS^t^, the growth response during a selected future period can be predicted.

In many children, information on heredity (parental heights), gestational age, birth size and/or pre-treatment growth are missing and are impossible to retrieve. For such children, a test-treatment year is often used to evaluate GH responsiveness. Therefore, a model that is accurate for use in patients with many different diagnoses of short stature is crucial as otherwise insufficient information precludes the use of any prediction model. The prediction model presented can be used in such children with the same degree of accuracy as the model that includes pretreatment data [2,5]. The model can also be used in a healthcare system preferring a year of test-treatment instead of pre-treatment investigations. However, for children in whom pre-treatment data *are *known, a model that uses pre-treatment data is preferable in order to optimize treatment during the first year and our suggestion is to use our previously published model to predict height gain during the first year [2,5] and when deciding whether or not to initiate treatment. The previously published model should also be used for selecting the GH dose for the first treatment years, as the younger the child is at the start of treatment, the better the response.

The variation in mean GH responsiveness between groups of children with different diagnosis is well known [20]. This has been the rationale behind published prediction models for use in different cohorts of children [1-5]. However, it is likewise well known that within every diagnostic group, there is great variation in growth response between individuals [21,22], as well as overlap between diagnostic groups. This continuum in GH responsiveness is the rationale for the inclusion of groups of children based on diagnosis in our work with the prediction of growth in response to GH, despite the fact that such cohorts are often defined based on arbitrary cutt-off values/statistics. Other evidence for the continuum in GH responsiveness is the overlap in growth responses using different GH doses within patients with the same diagnosis [3] and the wide range in GH dose needed to obtain a predefined serum insulin-like growth factor I (IGF-I) level [22]. The variation in GH dose needed to affect growth has previously been discussed in terms of misdiagnosis [23], but in recent years is more often seen as the expression of variations in individual responsiveness to GH [20,24,25]. In the present model, an individual factor, a Response Score, is expressed that also includes the effect of a fixed GH dose. In future, this factor should be investigated further and a dosing module added to the model.

## Conclusion

In conclusion, data are presented showing that the growth response to GH treatment can be described by a common algorithm for prepubertal non-syndromic children from different ethnic and geographic backgrounds, clinically classified as GHD/ISS, AGA/SGA and born later than 30 weeks of gestation. For each child, there is an individual parameter modulating the amplitude and timing of the response that depends on the child's GH responsiveness (constant) and treatment dose (here named Response Score). The observed first-year growth response to GH treatment reveals this value.

We provide a prediction model that is valid for the majority of short children worldwide in whom growth-supportive therapies are considered, despite the lack of historical auxological data on the child and without the need for any extensive investigations. The advantage of the present model is the 7-year reliability (i.e. the entire prepubertal period). The drawbacks are that an observed first-year GH growth response is needed and that the model is currently only validated for a GH dose close to 33 μg/kg/d. In order to increase the applicability of the mode, a collaborative study will be needed. This will be of importance in order to add a dose module for improved individualizing of the GH dose. Using this decision-making tool, it is possible to make reliable evidence-based decisions on whether, or how, to continue GH treatment in the individual child.

The model presented here can be used in non-syndromic short children, independently of birth size, and provides the best prediction accuracy available [1,3,4] (i.e. as high as previously obtained models using pretreatment information). The model can also serve as a tool for identifying those children who may benefit from long-term GH treatment, and will help to determine the GH dose needed during the first years of treatment in order to optimize individual catch-up growth.

## Abbreviations

AGA: appropriate for gestational age; AITT: arginine-insulin tolerance test; DIFF MPH SDS: difference in SDS between child and target height; GHD: growth hormone deficiency; IGF-I: insulin-like growth factor I; ISS: idiopathic short stature; RS: response score; SDS: standard deviation score; SDres: root mean square error of the residuals; SGA: small for gestational age.

## Competing interests

Kerstin Albertsson Wikland (KAW) declares that she had until 2005 an unrestricted research grant from Pharmacia/Pfizer. Berit Kristrom (BK) and Jovanna Dahlgren (JD) have received reimbursement for expert consultant work for Pfizer and JD has shares in the company. Aimon Niklasson (AN) has nothing to declare. Andreas FM Nierop (AFMN) works for Muvara, Multivariate Analysis of Industrial and Research Data Statistical Consultation, The Netherlands.

## Authors' contributions

BK, JD, AN, AFMN and KAW have all given substantial contribution to conception and design, analysis and interpretation of these data. AFMN has performed all of the modeling work. BK, JD, AN, AFMN and KAW have been involved in drafting the manuscript and have revised it critically for intellectual content. BK, JD, AN, AFMN and KAW have given final approval of the version to be published.

## Pre-publication history

The pre-publication history for this paper can be accessed here:

http://www.biomedcentral.com/1472-6947/9/1/prepub
